# Reduced Epithelial Na^+^/H^+^ Exchange Drives Gut Microbial Dysbiosis and Promotes Inflammatory Response in T Cell-Mediated Murine Colitis

**DOI:** 10.1371/journal.pone.0152044

**Published:** 2016-04-06

**Authors:** Daniel Laubitz, Christy A. Harrison, Monica T. Midura-Kiela, Rajalakshmy Ramalingam, Claire B. Larmonier, John H. Chase, J. Gregory Caporaso, David G. Besselsen, Fayez K. Ghishan, Pawel R. Kiela

**Affiliations:** 1 Department of Pediatrics—Steele Children’s Research Center, University of Arizona, Tucson, Arizona, United States of America; 2 Department of Biological Sciences, Center for Microbial Genetics and Genomics at Northern Arizona University, Flagstaff, Arizona, United States of America; 3 University Animal Care, University of Arizona, Tucson, Arizona, United States of America; 4 Department of Immunobiology, University of Arizona, Tucson, Arizona, United States of America; CWRU/UH Digestive Health Institute, UNITED STATES

## Abstract

Inflammatory bowel diseases (IBD) are associated with functional inhibition of epithelial Na^+^/H^+^ exchange. In mice, a selective disruption of NHE3 (*Slc9a3*), a major apical Na^+^/H^+^ exchanger, also promotes IBD-like symptoms and gut microbial dysbiosis. We hypothesized that disruption of Na^+^/H^+^ exchange is necessary for the development of dysbiosis, which promotes an exacerbated mucosal inflammatory response. Therefore, we performed a temporal analysis of gut microbiota composition, and mucosal immune response to adoptive T cell transfer was evaluated in Rag2^-/-^ and NHE3^-/-^/Rag2^-/-^ (DKO) mice with and without broad-spectrum antibiotics. Microbiome (16S profiling), colonic histology, T cell and neutrophil infiltration, mucosal inflammatory tone, and epithelial permeability were analyzed. In adoptive T cell transfer colitis model, *Slc9a3* status was the most significant determinant of gut microbial community. In DKO mice, NHE3-deficiency and dysbiosis were associated with dramatically accelerated and exacerbated disease, with rapid body weight loss, increased mucosal T cell and neutrophil influx, increased mucosal cytokine expression, increased permeability, and expansion of CD25^-^FoxP3^+^ Tregs; this enhanced susceptibility was alleviated by oral broad-spectrum antibiotics. Based on these results and our previous work, we postulate that epithelial electrolyte homeostasis is an important modulator in the progression of colitis, acting through remodeling of the gut microbial community.

## Introduction

It is well established that the vast assemblage of microbes harbored in the gastrointestinal tract exists in a mutualistic relationship with the host, which can become tenuous when disturbed [1–3]. Inflammatory bowel disease (IBD), an umbrella term for Crohn’s disease and ulcerative colitis, stems from a combination of precipitating environmental factors and genetic predisposition [4, 5]. IBD is associated with a dysregulated immune response to commensal gut-resident bacteria, and is one of several disorders associated with altered gut microbial ecology [6, 7]. The microbiota of IBD patients show profound shifts in the relative abundance of major and minor taxonomic groups, reflective of altered intestinal ecology [1, 8–10].

While the role of the microbiota has frequently been studied in the development of disease, the mechanisms by which the host’s inflammatory response itself affects the gut microbiota remain largely speculative [1, 11]. One of the non-immune consequences of mucosal inflammation that may result in changes within the microbial habitat is altered epithelial nutrient and especially electrolyte transport [12, 13]. Epithelial electrolyte balance is critical in regulating nutrient uptake, intestinal, intracellular and systemic pH, maintaining stool consistency, and in promoting normal gut motility, all of which may contribute to the maintenance of microbial equilibrium in health.

The apical epithelial Na^+^/H^+^ exchange system, represented by three isoforms, NHE2, NHE3, and NHE8, is of particular interest due to its role in transepithelial Na^+^ absorption and pH regulation. NHE3 in particular (encoded by the *SCL9A3* gene), provides a proton gradient which supports the function of other key carriers such as the butyrate-transporting H^+^-coupled electroneutral monocarboxylate transporter 1 (MCT1; *SLC5A8*), or proton-coupled oligopeptide carriers—PepT1, which plays a predominant role in the intestinal absorption of dipeptides and tripeptides, and small bacterially derived peptides, such as N-formylmethionyl-leucyl-phenylalanine, muramyl dipeptide, or L-Ala-[gamma]-D-Glu-meso-diaminopimelic acid (Tri-DAP). Most importantly, NHE3 expression and/or activity are depressed *in vitro* by IFNγ [14], *in vivo* in several models of experimental colitis [15–17], and in IBD patients [17–20]. Enteropathogenic bacteria inhibit [21, 22], whereas commensal *Lactobacillus acidophilus* upregulates intestinal NHE3 expression and function [23].

While these studies convincingly demonstrate the negative effects of inflammation or pathogenic bacteria on NHE3 function, we have previously described an inverse relationship whereby loss of NHE3 activity in NHE3^-/-^ mice lead to spontaneous, bacterially mediated distal colitis [24], greatly increased susceptibility to DSS-mediated epithelial injury [25], increased bacterial-epithelial adhesion, and changes in the mucous structure and enhanced bacterial translocation [24, 26]. Moreover, we and others have demonstrated that loss of NHE3 activity is associated with changes in ileal and colonic microbiota reminiscent of those described in IBD patients [27, 28]. However, the presence of T cells or gut microbiota is not imperative in the pathogenesis of DSS-induced colitis [29–31]. Considering the importance of T-cell driven immune response to commensal bacteria in the pathogenesis of human IBD, we sought to address whether loss of NHE3 activity modulates gut microbiota and mucosal immune responses in a chronic, progressive, T cell mediated [32], and microbiota-dependent [33], model of colitis. We found that in adoptive T cell transfer colitis, NHE3 deficiency and the associated dysbiosis dramatically accelerated and exacerbated colitis, increased mucosal CD4^+^ T cell and neutrophil homing, intestinal permeability, which was attenuated with broad-spectrum antibiotics. Dysbiotic microbiome was more strongly associated with NHE3 deficiency then with T cell mediated colitis *per se*. Na^+^/H^+^ exchange is, therefore, a crucial process for maintaining microbial homeostasis within the gut. Its disruption in IBD is likely a strong contributor to dysbiosis, which in turn affects the progression and severity of disease.

## Materials and Methods

### Ethics Statement

Animal protocol and procedures were approved by the University of Arizona Animal Care and Use Committee (approval # 07–126) and all experimental procedures were carried out in accordance with the approved guidelines.

### Animals

All animals were kept in a specific pathogen free barrier facility at the University of Arizona Bio-5 Institute. To generate Rag2^-/-^NHE3^-/-^ double knockouts (DKO), 129SvEv NHE3 heterozygotes were cross-bred with 129S6/SvEv Rag2 knockout mice (Taconic Farms). Rag2^-/-^NHE3^+/-^ mice were bred to generate DKO and Rag2^-/-^NHE3^+/+^ (Rag) littermates, which were used for further studies at 6–8 weeks of age. The barrier facility in the Bio-5 Institute at the University of Arizona used in this study provided a non-sterile environment with highly controlled procedures, including sterilization of all supplies and equipment entering the barrier and personnel working in barrier facilities required to wash their hands, wear gloves and sterilized clothing, including scrubs, shoes, caps, and masks. It provided *Helicobacter sp*-free and murine norovirus (MNV)-free environment. Sentinel mice were routinely monitored and determined as free from common murine pathogens (MHV, MPV, MVM, TMEV, *Mycoplasma pulmonis*, Sendai, EDIM, MNV, ectoparasites, and endoparasites). Mice were maintained in individually ventilated cages changed every two weeks. Sterile water and sterilized food were provided ad libitum. Breeders were fed Teklad Global 2019 diet and experimental mice were maintained on the NIH-31 modified open formula mouse/rat diet (7013). For antibiotic treatment experiments, mice were given a mixture of 200mg/l ciprofloxacin and 500mg/l metronidazole (Sigma-Aldrich, St. Louis, MO) in drinking water 7 day prior T cell (or PBS) injection and then continuously until tissue collection day, as we described previously [24]. Based on the average water consumption, approximate doses were 46.6 and 116 mg*kg^-1^*day^-1^ respectively. Experimental mice were sacrificed by CO_2_ inhalation followed by cervical dislocation.

### Adoptive T Cell Transfer

The following group designations are used throughout the manuscript: Rag/PBS and DKO/PBS–PBS-injected Rag2^-/-^ or Rag2^-/-^NHE3^-/-^ double knockout (DKO) mice, respectively; Rag/AT and DKO/AT—Rag2^-/-^ or Rag2^-/-^NHE3^-/-^ DKO mice adoptively transferred with 5x10^5^ cells in 500 μl PBS injected intraperitoneally. Naïve T cell transfer experiments were performed as described [32]. Briefly, 129S6/SvEv WT donors were sacrificed and spleens were extracted. Spleens were cut into small pieces, dissociated, and cells were passed through 100 μm strainer. After red blood cell lysis (Pharm Lyse, BD) CD4^+^ cells were purified using negative selection kit and magnetic columns (Miltenyi Biotec). Cells were fluorescently labeled (anti-mouse CD4-PE and anti-mouse CD45-FITC, BD) and CD4^+^CD45^RBhigh^ population was sorted using BD FACSAria IIu at the University of Arizona Flow Cytometry Core Facility. Cells were counted and a suspension of 10^6^ cells/ml was prepared. A solution of 5x10^5^ cells in 500 μl PBS was intraperitoneally injected into Rag2^-/-^ (Rag/AT) and DKO (DKO/AT) mice. Control groups were injected with sterile PBS (Rag/PBS and DKO/PBS). Stool was collected on the day of injection and every two days afterwards. Body weight was recorded at the same time and monitored closely thereafter.

### Histology and Immunohistochemistry

Colonic segments were collected, flushed with PBS and opened longitudinally on nitrocellulose membrane. For histological evaluation, tissues were fixed in 10% buffered formalin (Fisher Scientific), and embedded in paraffin. Samples were cut into 5 μm thick sections and hematoxylin-and-eosin (H&E) staining was performed. O.C.T embedded frozen segments were used for CD4 and neutrophil immunohistochemistry detection. Frozen samples were cut and immunoassayed using rat anti-CD4 (Novus Biologicals) or anti-mouse MCA771GA antibody recognizing a polymorphic 40 kDa antigen expressed by polymorphonuclear cells (AbD Serotec) and visualized using DAB Peroxidase Substrate Kit (Vector Laboratories).

### Intestinal Permeability

Intestinal permeability was assessed *in vivo* using FITC-labeled dextran (4 kDa, Sigma) as a mucosal tracer flux marker. Prepared solution in PBS was administrated by gastric gavage at the final dose 60 mg / 100g body weight. 4 hours later, mice were sacrificed and blood samples were taken on heparin by cardiac puncture. The fluorescence in the resulting plasma was measured in 96 well plate using SpectraMax M3 plate reader (ex485 / em525) and the concentration of FITC-label marker in the blood was calculated against a respective standard curve.

### Flow Cytometry

The mesenteric lymph nodes (MLNs) were collected and dissociated with frosted glass slides. Cells were passed through 100 μm strainer and stained with FITC-conjugated anti-CD4 and APC-conjugated anti-CD25. Cells were then fixed, permeabilized and stained with PE-conjugated anti-FoxP3 antibody. Data was collected using the LSRII FORTESSA operated by FACSDiva software followed by raw data analysis with FlowJo v. 7.6.5 software.

### Fecal DNA Extraction

Fecal pellets were collected from mice and stored at -80°C. Fecal DNA was extracted using a phenol-chloroform extraction protocol described by the CBDM Laboratory (Harvard Medical School, Boston, MA). Briefly, frozen fecal specimens were incubated at room temperature for 2 hours with 500 μl of 0.1 mm zirconium beads (BioSpec Products) in 710 μl Lysis Buffer containing 100mM NaCl, 10mM Tris, 100mM EDTA, 0.2 mg/ml Proteinase K and supplemented with DNAse-free 5.9% SDS (Fisher Scientific) solution. 500 μl phenol:chloroform:isoamyl alcohol (24:24:1) reagent was added to each sample followed by cell disruption for 2 minutes using Mini bead-beater (Biospec Products). Then, samples were centrifuged (5,900 x g, 3 min, 4°C) and the aqueous phase was transferred into a new tube and again extracted with 300 μl of phenol:chloroform:isoamyl alcohol. After centrifugation (15,700 x g, 3 min, 4°C) the aqueous phase was collected into a new tube and DNA was precipitated with equal volume of (-20°C) isopropyl alcohol and 1:10 (v/v) 3M sodium acetate. Samples were incubated on ice, pelleted by centrifugation (15,700 x g, 20 min, 4°C), rinsed with cold ethanol, spun and dried. Purified DNA was resuspended in 200 μl TE buffer (Amresco) and quantified on Nanodrop ND-1000. DNA samples were stored at -80°C.

### Bacterial Quantitative qPCR

*Firmicutes*, Clostridia cluster IV, and Clostridia cluster XIVa were amplified from 10 ng fecal DNA using PerfeCTa SYBR Green Fastmix (Quanta Biosciences) at primer annealing temperatures optimized by melting curve. Cq values were used to calculate relative abundance. All samples were normalized to universal 16S control, and all reactions were done on a CFX96 Real-Time System (BioRad). Primers were selected from De Gregoris et al [34] and ordered from Sigma. Sequences [35] and annealing temperatures can be found in Table 1.

**Table 1:** Primers used for quantitative real-time PCR analysis of respective taxa. F–forward, R- reverse. Annealing temperature for the 3-step PCR protocol is indicated for each primer pair.

Bacterial Group	Primer Name	Primer Sequence	Annealing Temp (°C)
*Firmicutes*	928F-Firm	F 5’-TGAAACTYAAAGGAATTGACG	55.7
	1040FirmR	R 5’-ACCATGCACCACCTGTC	
*Clostridia IV*	CL-IV-F	F 5’-CCTTCCGTGCCGSAGTTA	52.8
	CL-IV-R	R 5’-GAATTAAACCACATACTCCACTGCTT	
*Clostridia XIVa*	CL-XIVa-F	F 5’-AAATGACGGTACCTGACTAA	57.0
	CL-XIVa-R	R 5’-CTTTGAGTTTCATTCTTGCGAA	
Universal 16S	926F	F 5’-AAACTCAAAKGAATTGACGG	59
	1062R	R 5’-CTCACRRCACGAGCTGAC	

### Gut Microbiome Analysis

The hypervariable V4 region of the 16S rRNA gene was amplified from each sample using barcoded 806R primers and 515F primer [36] and 5 Prime Hot MasterMix (5 Prime, Germany) in triplicates. Quality of the amplicons and potential contaminations were checked on an agarose gel. Amplicons were quantified using Picogreen (Invitrogen), according to the manufacturer’s protocol. 240ng of DNA from each sample were pooled and cleaned using UltraClean PCR Clean-Up Kit (MoBio). Pooled amplicons were diluted, denatured (0.2N NaOH) and sequenced on MiSeq platform (Illumina) using custom primers [36]. Due to the limited sequence diversity among 16S rRNA amplicons, 10% of the PhiX control library (Illumina) made from phiX174 was added to the run. Final concentration of 6.75pM of the pooled 16S rRNA library was subjected to the paired-end sequencing using 2 x 150bp MiSeq Reagent Kit V2 (Illumina). Sequencing of all samples collected from the mice was performed at Argonne National Laboratories on Illumina MiSeq (SN M02149, with the MiSeq Control Software v 2.2.0). The run of total 389 pooled samples generated 13,322,438 sequences. After de-multiplexing and quality filtering, 12,001,960 reads remained and with a median length of 253 bases. Out of 389 pooled samples 128 samples from this study with total number of reads 3,839,752 were taken for analysis presented in this manuscript. Other samples belong to different experiment and are not discussed here.

De-multiplexing and filtering was done using QIIME 1.9.1 software package [37]. Sequences were assigned to operational taxonomic units (OTU) with a 97% similarity threshold using QIIME’s uclust-based open-reference OTU picking protocol against SILVA reference database (release 119, http://www.arb-silva.de/download/archive/qiime/). Sequences that did not match the reference database were clustered *de novo*, thus all sequences were included in the analysis. The average sequence numbers per sample was 29,998.1 ± 8,075.3 (mean ± SD). The samples with the number of sequences lower than 17,900 were filtered out, which resulted in the exclusion of 4 samples. Core diversity analysis was performed on the OTU tables including alpha and beta diversity as well as taxonomic summary as implemented in QIIME software package. All sequence data generated in this study will be available in Qiita (formerly called the QIIME DB) as Study ID 10309 (https://qiita.ucsd.edu/study/description/10309) and in Open Science Framework (DOI 10.17605/OSF.IO/UWFAP, https://osf.io/uwfap/).

### Functional Profiling of the Microbial Community

PICRUSt (release 1.0.0) was used to predict functional profiling of the microbial communities based on the 16S rRNA gene sequences [38]. All sequences from each sample were searched against the Greengenes (gg_13_5) at the 97% identity (closed OTU picking method). The OTU tables were normalized by dividing each OTU by the known/predicted 16S rRNA gene copy number abundance, and the prediction of the metagenome functional content was done using the KEGG Orthology (KO) classification scheme. The predicted metagenome BIOM table was analyzed and visualized using the Statistical Analysis of Taxonomic and Functional Profiles (STAMP) software package v. 2.0.9.

### Quantitative RT-PCR

Colonic gene expression of the selected genes in all experimental and control groups were analyzed independently by qPCR. RNA was extracted from distal and proximal colonic tissues with TRIzol reagent (Invitrogen, Carlsbad, CA). 250ng of total RNA was reverse-transcribed using the cDNA synthesis qScript (Quanta Biosciences) or Transcriptor Universal cDNA Master kit (Roche). qPCR reactions for specific genes of interest as well as endogenous reference gene were set up using commercially available TaqMan primers (Applied Biosystems), PerfeCTa qPCR Super Mix (Quanta Biosciences) and cDNA (10% of the real-time reaction). Data were analyzed and expressed as a relative fold change of the gene expression, normalized to the endogenous reference (GAPDH) gene and relative to normalized Cq value obtained from control group (2^-ΔΔCt^) as indicated.

### Statistical Analysis

Statistical analyses of non-sequencing data were performed using Prism 6 (GraphPad Software Inc.) or STAMP 2.0.9 package for functional prediction of the microbial community. All data were tested for normal distribution of the data by the Shapiro-Wilk test. Data sets that passed the normality test were analyzed using one-way ANOVA with Fisher’s LSD post-hoc test. Those that did not pass the normality test were analyzed using Kruskal-Wallis test with Dunn’s multiple comparisons test.

## Results

### At Baseline, NHE3^-/-^ and NHE3^-/-^Rag2^-/-^ DKO Mice Develop Comparable Spontaneous Colonic Inflammation

**Fig 1:**
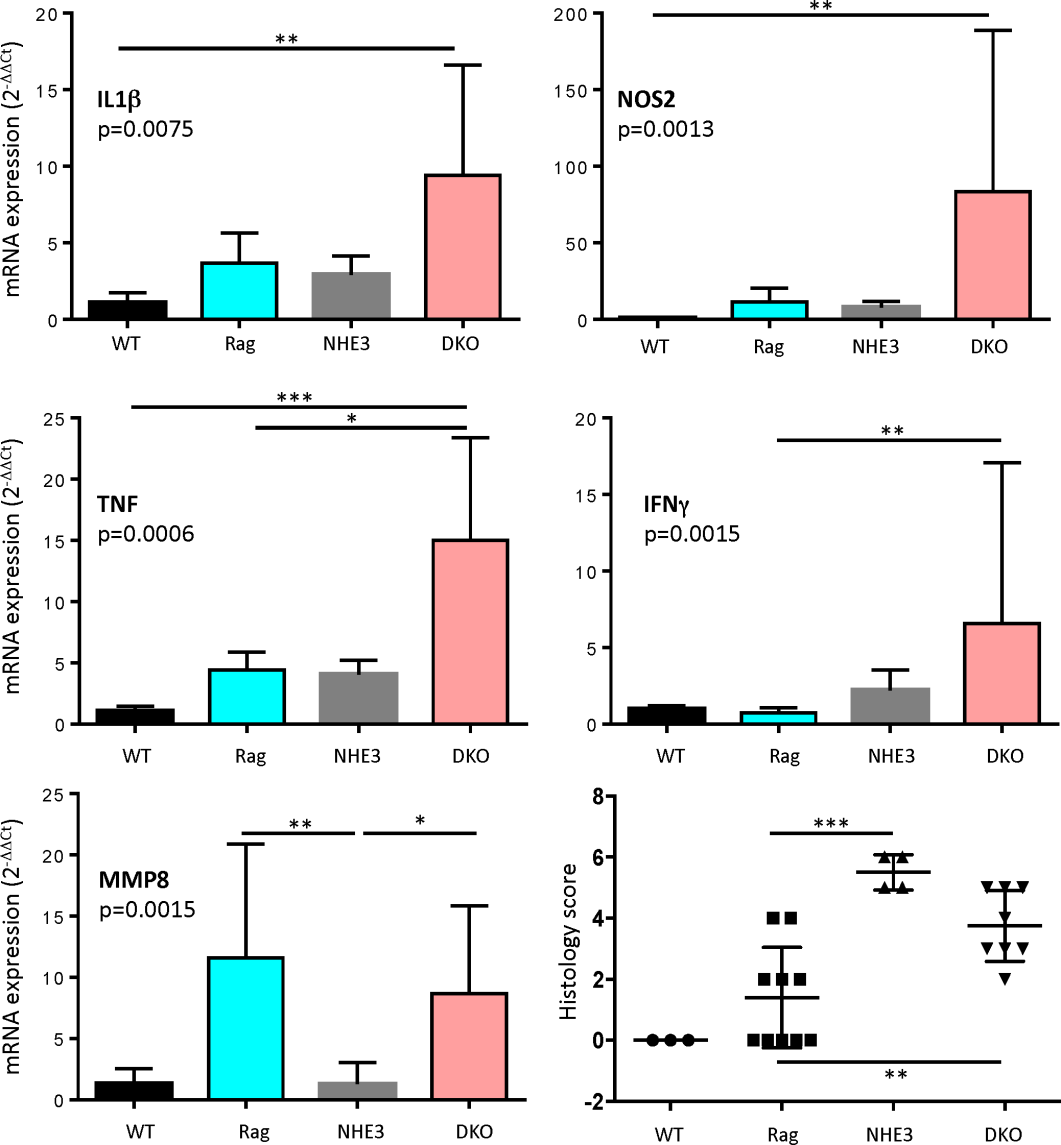
Cytokine expression and histological soring in distal colon in WT (n = 3), Rag2^-/-^ (n = 10), NHE3^-/-^ (n = 5) and Rag/NHE3 DKO mice (n = 9). Bar graphs represent mean with SD values; dots represent individual mice and horizontal lines are mean values ± SD. Statistical analysis were performed using Kruskal-Wallis test followed by Dunn’s multiple comparisons test. Asterisks in all panels indicate statistical significance: * P < 0.05, ** P < 0.01, *** P< 0.001, **** P < 0.0001.

We have previously shown that a change in microbial environment via re-derivation of NHE3-deficient mice from conventional to a barrier SPF facility results in reduced colitis, and re-conventionalization partially restores the original inflammatory phenotype [27]. For this study, we crossed NHE3^-/-^ with Rag2^-/-^ mice (DKO) maintained in the barrier facility to generate T- and B-cell deficient hosts with reduced epithelial Na^+^/H^+^ exchange. In order to determine baseline inflammatory response between groups before proceeding with adoptive transfer, we assessed the degree of distal colitis in NHE3^-/-^ and DKO strains. While NHE3^-/-^ mice develop distal colitis primarily via activation of innate immune mechanisms without a significant engagement of effector T cells [24], loss of B and T lymphocytes, and presumably the associated loss of regulatory T cells (Treg), led to higher mucosal expression TNFα, IFNγ, IL1β, and NOS2 in the distal colon of DKO mice, as compared with WT, Rag2^-/-^, or NHE3^-/-^ strains (Fig 1). This increase was not consistent among the DKO mice, and despite a clear trend, it did not reach statistical significance when compared to NHE3^-/-^ mice. Rag2^-/-^ and DKO mice had similarly elevated mucosal expression of MMP8, a neutrophil collagenase and a surrogate marker of neutrophilic infiltration (Fig 1), consistent with the reported increase in neutrophil infiltration in the colonic lamina propria of Rag^-/-^ at baseline [39]. Mean pathology score was lower in DKO than in NHE3^-/-^ mice, albeit due to considerable deviation, it did not reach significance.

### Accelerated and Exacerbated T Cell Transfer Colitis in NHE3^-/-^Rag2^-/-^ DKO Mice Is Alleviated by Broad-Spectrum Antibiotics

**Fig 2:**
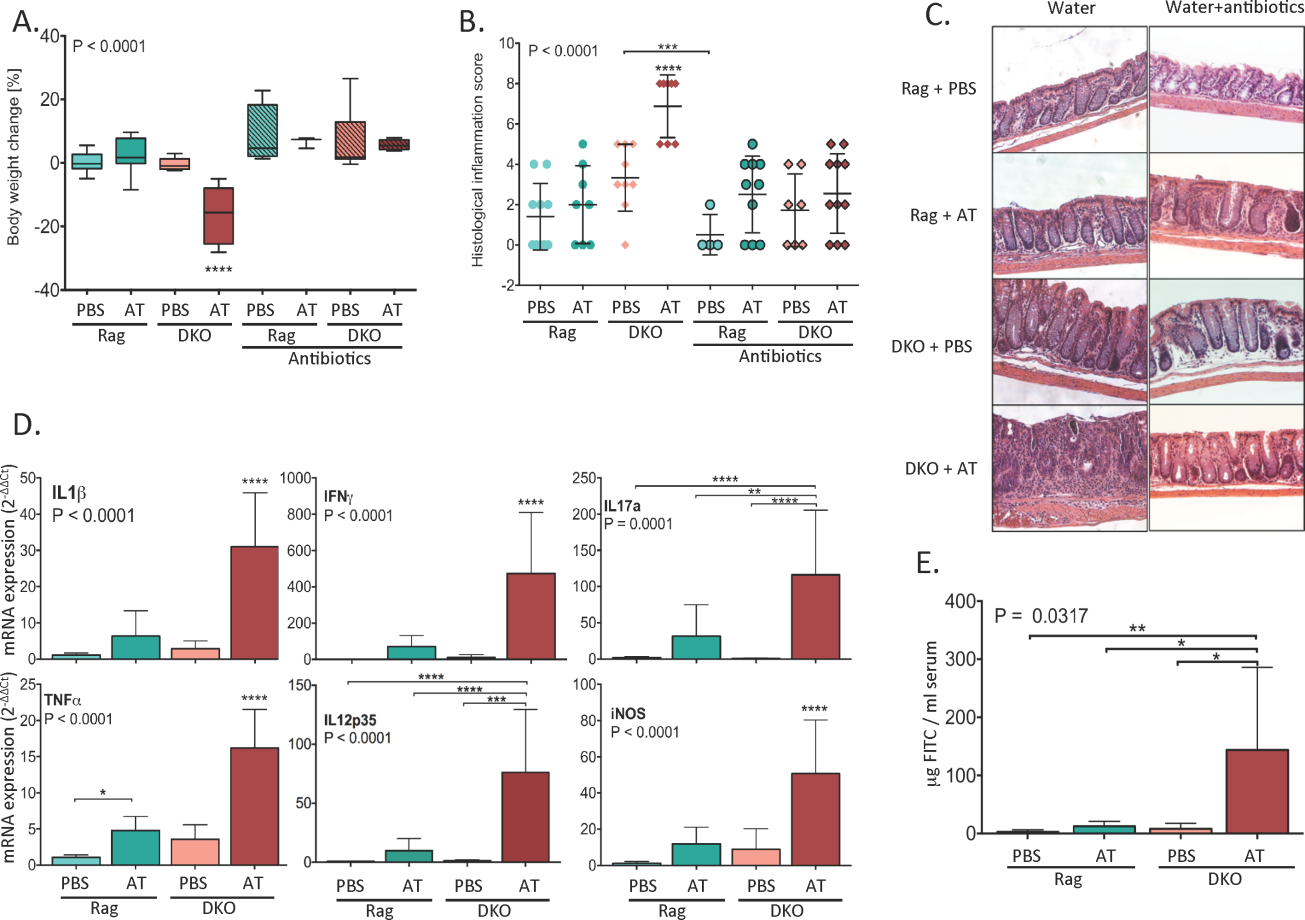
After adoptive T cell transfer, NHE3^-/-^Rag2^-/-^ DKO mice develop severe colitis, which is alleviated by broad-spectrum antibiotic treatment. (**A**) Changes in body weight (% change of initial value 13 days post-transfer; boxes extend from 25th to 75th percentiles of values, whiskers represent min and max value, line within the box represents the mean value); (**B**) Histological colitis scoring (dots represent individual mice and lines mean value ± SD); (**C**) Representative H&E images of distal colon from respective experimental groups; (**D**) qPCR analysis of mucosal expression of proinflammatory cytokines in Rag/PBS (N = 10), Rag/AT (N = 8) and DKO/PBS (N = 9) and DKO/AT (N = 8) mice (bar graphs represent mean ± SD values); (**E**) Intestinal mucosal permeability measured by FITC-labeled dextran tracer flux (N = 3–6). Statistical analysis was performed using one-way ANOVA (ANOVA P value is indicated in each panel) with Fisher’s LSD post-hoc test. Asterisks in all panels indicate statistical significance: * P < 0.05, ** P < 0.01, *** P< 0.001, **** P < 0.0001. Brackets point to differences between indicated groups. Asterisks without brackets indicate a statistical difference from all other groups within the panel. Each experimental groups is color coded in a way consistent in the remaining figures.

In order to determine whether reduced epithelial Na^+^/H^+^ exchange altered susceptibility to T cell-mediated colitis, we adoptively transferred Rag2^-/-^ and DKO mice with naïve T cells. Following adoptive transfer of naïve CD4^+^CD45RB^hi^ T cells, Rag2^-/-^ mice typically develop mild to moderate colitis within 6–8 weeks. In DKO mice, however, critical moribundity (body weight loss ≥ 20% of initial body weight) was reached 13 days following T cell transfer (Fig 2A), at which point the mice were sacrificed and the last stool samples along with tissue samples were acquired for analysis. With the exception of modest, but significant increase of mucosal TNFα expression, 13 days post-transfer Rag2^-/-^ mice did not develop overt signs of colitis (Fig 2). Substantial body weight loss in T-cell transferred DKO mice was accompanied by a significant increase in the histological inflammation score in the colon (Fig 2B and 2C), and a dramatic increase in mucosal expression of IL1β, IFNΥ, IL17A, TNFα, IL12p35, and iNOS (Fig 2D). At day 13 post-transfer, epithelial barrier breach was evident in DKO mice, as demonstrated by mucosal flux of FITC-labeled dextran (Fig 2E). Combination of two broad-spectrum antibiotics (ciprofloxacin plus metronidazol) used commonly in IBD clinical practice [40], prevented the development of colitis in DKO mice (Fig 2A–2C), thus suggesting that impaired epithelial Na^+^/H^+^ exchange, a process also observed in human IBD patients, dramatically increases susceptibility to experimental T cell-mediated colitis in a microbiome-dependent manner.

### Mucosal T Cell and Neutrophil Homing Are Dramatically Increased in NHE3^-/-^Rag2^-/-^ DKO Mice after Adoptive T Cell Transfer

**Fig 3:**
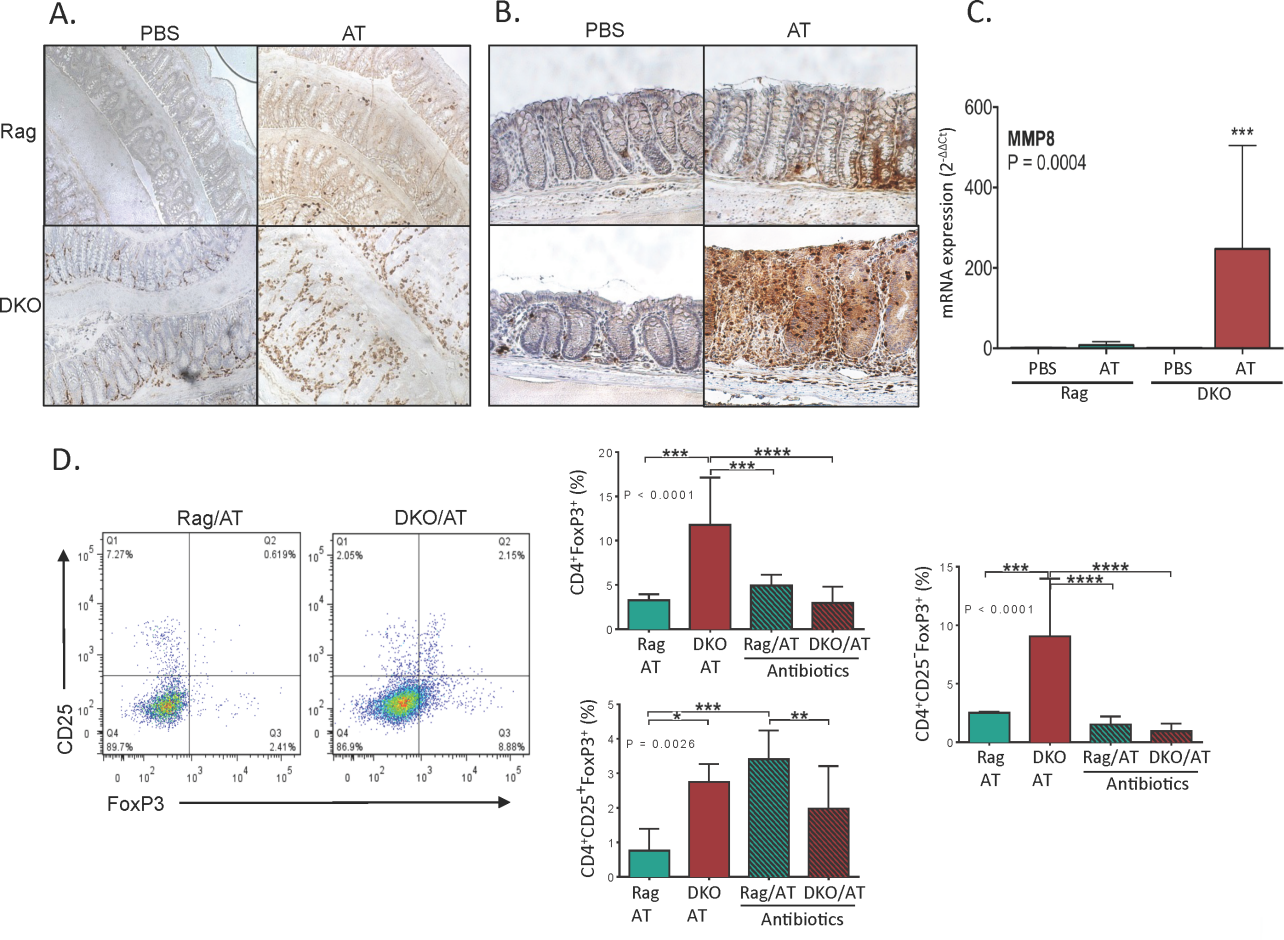
Increased T cell and neutrophil infiltration in the colonic mucosa of DKO/AT mice is accompanied by altered MLN Treg phenotype. (**A and B**) Representative images of immunohistochemical analysis of (**A**) CD4^+^ cell abundance and (**B**) neutrophil abundance in the distal colonic mucosa; (**C**) qPCR analysis of mucosal expression of neutrophil collagenase MMP8; (**D**) Flow cytometry analysis of regulatory T cells in mesenteric lymph nodes (MLN) in adoptively transferred mice and effect of antibiotics. Bar graphs represent mean with SD values. Statistical analysis were performed using one-way ANOVA (P value indicated in each panel) with Fisher’s LSD post-hoc test. Asterisks in all panels indicate statistical significance: * P < 0.05, ** P < 0.01, *** P< 0.001, **** P < 0.0001.

Infiltrating T cells and neutrophils are key effectors of mucosal damage in IBD. Thirteen days after adoptive T cell transfer, colonic mucosa of Rag2^-/-^ mice showed limited and scattered T cell infiltration, and limited and focal granulocyte infiltration (Fig 3A and 3B). We observed increased number of CD4^+^ cells in PBS-treated DKO mice, primarily concentrated around the base of the crypts (Fig 3A). These likely represent innate immune cells such as monocytes, macrophages, dendritic cells, or activated NK cells, all of which can express CD4. In response to T cell transfer, DKO mice responded with a significantly greater influx of both immune cell populations with uniform trans-mucosal infiltrates of CD4^+^ cells and granulocytes (Fig 3A and 3B). Neutrophil immunohistochemistry was further confirmed by mucosal expression of MMP8, which in some cases exceeded 500-fold increase in T cell transferred DKO mice as compared to PBS-injected or T cell transferred Rag2^-/-^ mice (Fig 3C).

Flow cytometry analysis of mesenteric lymph node (MLN) T cells showed similar degree of T cell activation in adoptively transferred Rag2^-/-^ and DKO strains, based on CD44 and CD69 cell surface expression (data not shown). Regulatory T cell (Treg) analysis showed a significant increase in the total CD4^+^FoxP3^+^ cell pool in T cell transferred DKO mice compared to Rag2^-/-^ mice (11.77+5.34 vs. 3.26%±0.68, respectively; Fig 3D). While this included a modest expansion of the classical immunosuppressive CD4^+^CD25^+^FoxP3^+^ iTregs, of the 11.77% CD4^+^FoxP3^+^ Tregs observed in DKO mice, the majority (9.03%+4.9) were CD25-negative (CD4^+^CD25^-^FoxP3^+^)–significantly more than in Rag2^-/-^ mice (Fig 3D). This population has been recently reported in several human autoimmune diseases and in experimental ileitis (see Discussion). Interestingly, antibiotics, which prevented the development of colitis, suppressed the expansion of total FoxP3^+^ and CD25^-^FoxP3^+^ Tregs, thus indicating that this CD25-negative Treg population expands as a consequence of inflammation and/or dysbiosis (Fig 3D). Antibiotic treatment increased the relative contribution of CD25^+^FoxP3^+^ Tregs in T cell transferred Rag2^-/-^ mice, but to a significantly lesser degree in DKO mice (Fig 3D).

### Significant Colonic Dysbiosis in NHE3^-/-^Rag2^-/-^ DKO Mice at Baseline Is Not Further Modulated by Aggravated Response to Adoptive T Cell Transfer

Since aggravated colitis in DKO mice was ameliorated with broad-spectrum antibiotics, and thus clearly depended on the gut microbiota, we sought to determine the differences in the composition of the gut microbiome between Rag2^-/-^ and DKO mice. We regularly collected fecal samples from mice that had been injected PBS or with naïve T cells, and subsequently amplified and sequenced the V4 region of 16S rRNA gene. A total of 3,839,752 sequence reads were obtained from 128 samples (average 29,998 ± 8,075.3 reads/sample (mean ± SD)). These sequences were clustered into OTUs, and all reads with less than 97% sequence similarity with a reference sequence in the SILVA database. Representative sequences for each OTU were assigned to respective taxonomic levels by comparing against the SILVA database.

**Fig 4:**
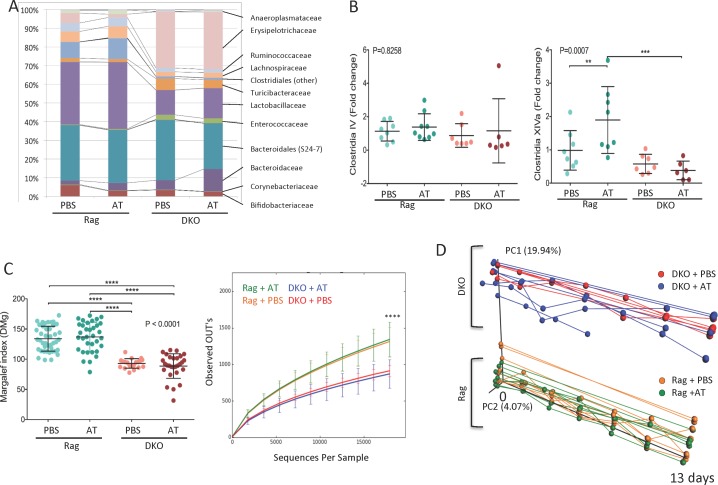
Changes in microbiota composition in Rag and DKO mice before and after adoptive T cell transfer. (**A**) Endpoint taxonomic analysis (day 13) of the relative abundance of bacterial families in fecal samples; (**B**) Endpoint qPCR analysis of selected clusters of *Clostridia*; (**C**) Endpoint analysis of alpha diversity (Margalef index and the number of observed OTUs); (**D**) Principal Coordinate Analysis (PCoA) of unweighted UniFrac distances between of microbiota samples (first two principal coordinate axes versus time shown) in Rag2^-/-^ and DKO mice with or without adoptive T cell transfer over the course of the experiment. Samples were collected every 2 days post injection. Prematurely ended lines (DKO+AT) represent mice sacrificed earlier due to a critical body weight loss (≥20%). Points represent individual mice. In **B**-**C**, statistical analyses were performed using one-way ANOVA (P value indicated in each panel) with Fisher’s LSD post-hoc test. Asterisks in all panels indicate statistical significance: * P < 0.05, ** P < 0.01, *** P< 0.001, **** P < 0.0001.

**Fig 5:**
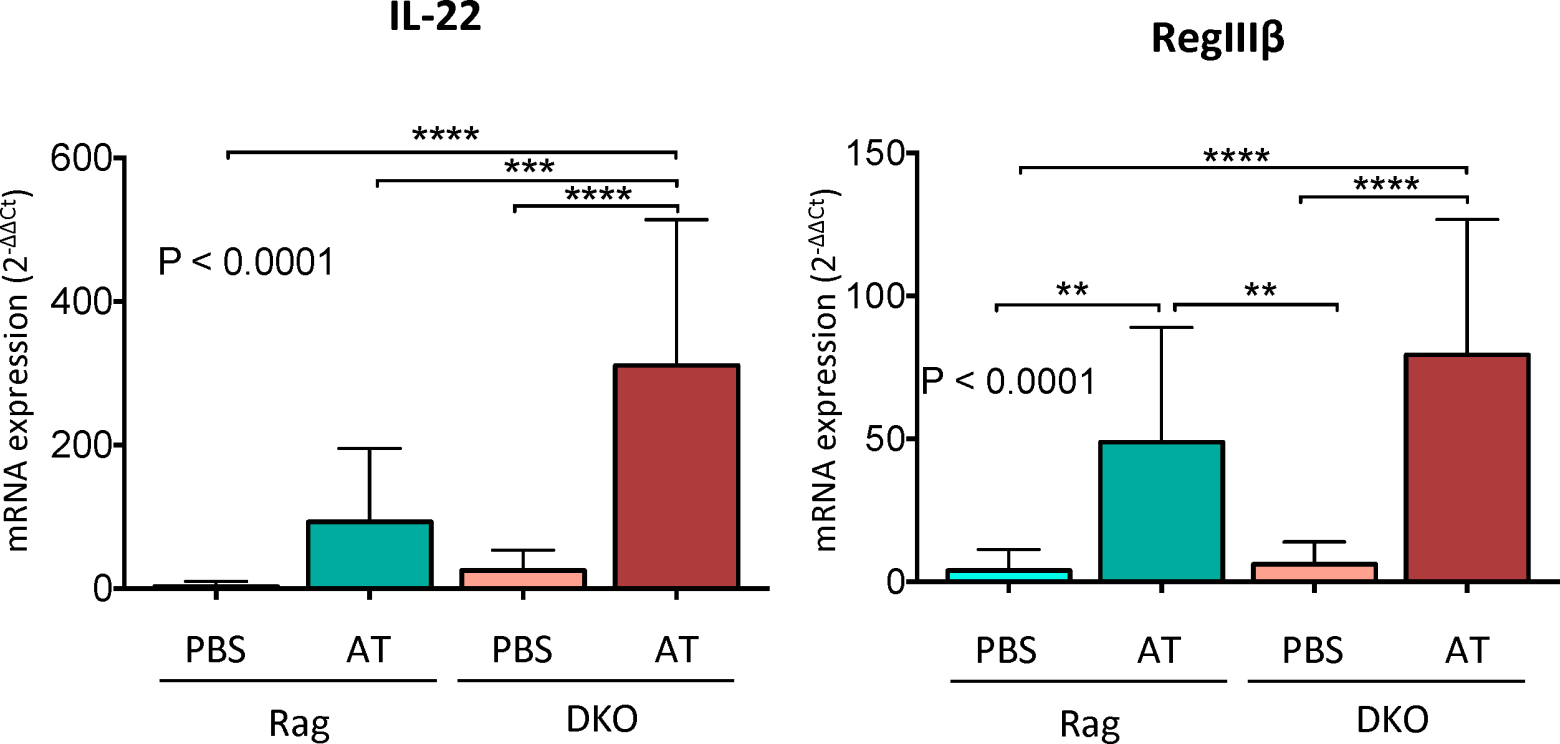
Mucosal expression of protective cytokine IL-22 and IL-22-regulated antimicrobial peptide, RegIIIβ. Bars represent mean values ± SD. Statistical analysis were performed using one-way ANOVA (P value indicated in each panel) with Fisher’s LSD post-hoc test. Asterics in all panels indicate statistical significance: ** P < 0.01, *** P< 0.001, **** P < 0.0001.

Regardless of treatment (PBS or T cell transfer), NHE3-deficiency in DKO mice was associated with differences in the relative abundance of several taxa at the confidence level of 99.5%. A dramatic loss of *Lactobacillaceae* and *Clostridiales* was observed in DKO compared to Rag2^-/-^. Other examples include reduced relative abundance of the *Lachnospiraceae*, *Ruminococcaceae*, *Anareoplasmataceae*, *Coriobacteriaceae*, *Mogibacteriaceae* and *Dehalobacteriaceae* families. Lower abundance of *Bifidobacteriaceae* was also observed, albeit without reaching a statistical significance. DKO mice had a dramatically higher relative abundance of *Erysipelotrichaceae* compared to Rag2^-/-^, as well as elevated *Bacteroidales (S24-7)*, *Turicibacteraceae*, *Enterococcaceae* and *Bacillaceae* (Fig 4A, Table 2). The expansion of *Erysipelotrichaceae* in DKO mice was predominantly due to a prominent increase in the relative abundance of the *Allobaculum* genus. Expansion of this genus has also been reported in experimental colitis in IL-22 deficiency model associated with defective production of mucosal antimicrobial peptides RegIIIβ and RegIIIγ [41]. However, compared to T-cell transferred Rag^-/-^ mice, DKO mice had significantly higher mucosal expression of IL-22 and RegIIIβ (Fig 5).

**Table 2:** Taxonomic analysis of relative family abundance. Family level relative abundance [%] differences in Rag2^-/-^ vs. DKO treated with PBS (+PBS) or after adoptive T cell transfer (+AT). Fecal sample collected and pooled from these groups were used in the FMT experiment. Asterisks indicate significant differences in Dunn’s test between respective experimental groups; * p<0.05, ** p<0.01, *** p<0.001, **** p<0.0001 (ns–no significant difference).

Family	Rag+PBS [%]	Rag+AT [%]	DKO+PBS [%]	DKO+AT [%]	Kruskal-Wallis test with Dunn’s multiple comparison test				
					Rag+PBS vs DKO+PBS	Rag+PBS vs Rag+AT	DKO+PBS vs DKO+AT	Rag+ AT vs DKO+AT	Anova P value
Lactobacillaceae	33.46	35.71	13.21	16.18	****	ns	ns	****	<0.0001
Bacteroidales (S24-7)	29.63	28.33	32.36	24.49	ns	ns	ns	ns	0.0864
Clostridiales other	8.59	11.05	1.05	1.02	****	ns	ns	****	<0.0001
Bifidobacteriaceae	5.97	2.95	3.40	2.54	ns	**	ns	ns	0.0023
Lachnospiraceae	5.48	6.20	2.44	2.79	***	ns	ns	****	<0.0001
Erysipelotrichaceae	5.29	1.67	30.06	30.62	****	ns	ns	****	<0.0001
Ruminococcaceae	4.60	4.74	2.04	1.70	****	ns	ns	****	<0.0001
Bacteroidaceae	2.10	3.98	5.05	11.91	**	**	ns	ns	<0.0001
Turicibacteraceae	2.03	1.67	6.17	4.44	****	ns	ns	***	<0.0001
Anaeroplasmataceae	1.46	2.15	0.29	0.66	****	ns	ns	**	<0.0001
Enterococcaceae	0.35	0.43	2.54	2.48	****	ns	ns	****	<0.0001
Coriobacteriaceae	0.35	0.30	0.14	0.16	***	ns	ns	**	<0.0001
[Mogibacteriaceae]	0.08	0.09	0.00	0.00	****	ns	ns	****	<0.0001
Dehalobacteriaceae	0.04	0.05	0.00	0.01	****	ns	ns	****	<0.0001
Christensenellaceae	0.03	0.02	0.04	0.02	ns	ns	ns	ns	0.0294
Streptococcaceae	0.02	0.02	0.03	0.03	ns	ns	ns	ns	0.9306
Bacillales other	0.02	0.02	0.07	0.04	****	ns	ns	**	<0.0001
Lactobacillales other	0.01	0.01	0.01	0.01	ns	ns	ns	ns	0.0054
Bacillaceae	0.01	0.01	0.07	0.06	****	ns	ns	***	<0.0001
Corynebacteriaceae	0.01	0.01	0.01	0.01	ns	ns	ns	ns	0.4094
Clostridiaceae	0.00	0.11	0.00	0.05	ns	ns	*	ns	0.0141

Within 13 days, adoptive T cell transfer caused only minor changes in the taxonomic group prevalence in both Rag2^-/-^ and DKO mice. In Rag2^-/-^, we observed decreased relative abundance of *Bifidobacteriaceae* and expansion of *Bacteroidaceae*, while in DKO mice, only small increase in *Clostridiaceae* reached significance (Table 2). Among order *Clostridiales*, clusters IV and XIVa are known to play an influential role in gut homeostasis and the induction of iTregs, particularly through butyrate production. Among *Clostridiales*, cluster XIVa (but not IV) increased in relative abundance in T-cell transferred Rag2^-/-^, but was significantly decreased in DKO mice post-transfer (Fig 4B). Relative abundance of segmented filamentous bacteria (SFB) did not change in DKO mice at baseline or by adoptive transfer of naïve T cells, as determined by qPCR (data not shown). NHE3 deficiency was associated with a significant decrease in microbial alpha diversity, as measured by the Margalef index and OTU count number. Alpha diversity was not further influenced by T-cell induced inflammation (Fig 4C).

**Fig 6:**
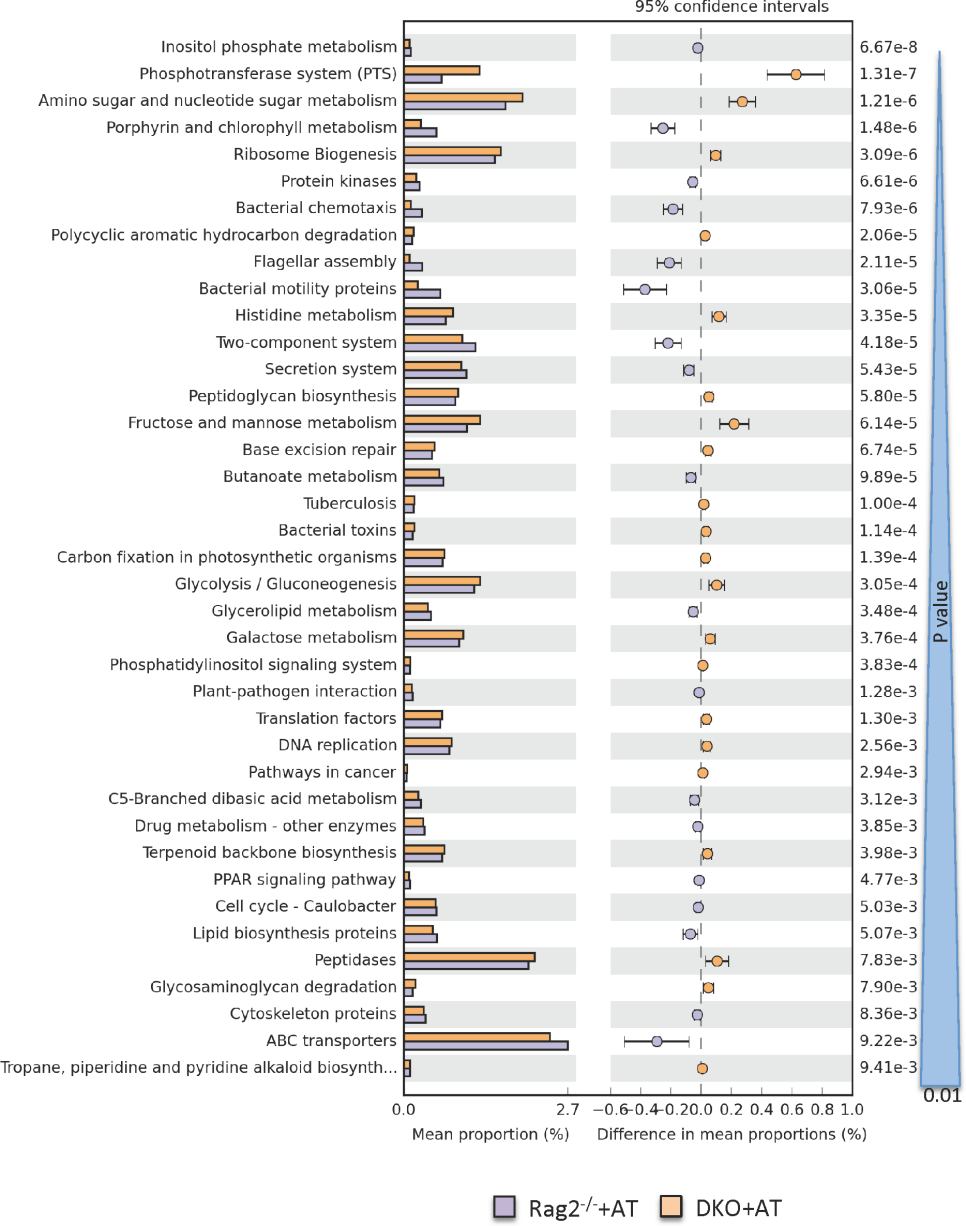
PICRUSt prediction of functional profiling of the microbial communities based on the 16S rRNA gene sequences. Extended error bar plot indicating differences in functional profiles of the Rag+AT and DKO+AT microbiota (at taxonomic Level 3). All unclassified reads were removed and categories with minimum of 20 reads, and P value greater than 0.01 is displayed. Categories are sorted by P value calculated using two-sided Welch’s t-test.

Principal Coordinate Analysis (PCoA) of unweighted UniFrac distances [42] showed differences between Rag2^-/-^ and DKO mice gut microbiome composition from the onset of the study and throughout the experiment (Fig 4D). Similar to taxonomic analysis (Fig 4A), this beta diversity analysis indicated that inflammation triggered by adoptive T cell transfer did not significantly change the microbiome further within the 13-day time frame of the study (Fig 4D). This, along with the Analysis of Similarity (ANOSIM, Table 3), indicates the major factor shaping microbiome composition was almost entirely related to the NHE3-deficiency in DKO mice. These data corresponded with the PICRUSt analysis of the functional profiling of the microbial community as visualized and analyzed using STAMP software package. Functional annotation of the fecal microbiome of Rag2^-/-^ and DKO mice with adoptive T cell transfer was consistent with potentially pro-inflammatory enterotype (Fig 6). Collectively, this set of data implies that loss of NHE3 activity leads to considerable dysbiosis, which may be responsible for baseline inflammation as well as a dramatic response to adoptively transferred naïve T cells.

**Table 3:** Summary of beta diversity analysis. A non-parametric ANOSIM (Analysis of Similarity) method was applied to dataset presented in Fig 3D (PBS or adoptive T cell transfer in Rag2^-/-^ or DKO mice, from day 0 to day 13). Among single variables, only the genotype of the T cell recipient mice was significantly different based on a UniFrac beta diversity metric. In multivariate analysis, genotype was the single contributor to differences in beta diversity. Each row is a comparison of microbial diversity done with one or more factors, indicated by the labels. Beta diversity was calculated using Analysis of Similarity (ANOSIM) and significance was considered reached when both a and b were true as follows: a) p-value < 0.05 and b) R value > 0.3.

Genotype	Treatment	Time	R	p value	Significance beta diversity
**Genotype**			0.967	0.001	Yes
	Treatment		0.0259	0.028	No
		Time	-0.0173	0.768	No
**Genotype**	Treatment		0.65	0.001	Yes
**Genotype**		Time	0.503	0.001	Yes
	Treatment	Time	-0.023	0.801	No

## Discussion

While the general concept of contribution of commensal gut microbiome to the pathogenesis of aberrant immune response in IBD has been firmly established, it remains debatable whether the changes in microbial diversity and composition are the cause or the result of the inflammation. Similarly, the specific driving forces leading to gut microbial dysbiosis in IBD remain unknown, largely due to experimental difficulties in dissociating these factors from the inflammatory process itself. We hypothesized that altered epithelial nutrient transport, especially the reduced Na^+^/H^+^ exchange activity, may be a significant contributor to inflammation-associated microbial dysbiosis which results in further disease exacerbation. In our previous work, we showed that NHE3 deficiency in mice is associated with a susceptibility to dextran sulfate (DSS)-induced mucosal injury [25]. However, DSS-induced colitis does not require the presence of gut microbiota and a more severe form of disease can be induced in germ-free mice, suggesting a protective role of bacteria in this model [29, 30]. Considering the importance of T-cell driven immune response to commensal bacteria in the pathogenesis of human IBD, we aimed at testing the role of epithelial Na^+^/H^+^ exchange in a model in which mucosal inflammation is initiated by CD4^+^ T cells, and is reliant on gut microbiota [33], as more representative of progressive human IBD [32]. Here, we used mice genetically deficient in one of the apical Na^+^/H^+^ exchangers, NHE3, to demonstrate the consequences of reduced epithelial Na^+^/H^+^ exchange on the colonic microbiota and the severity of T-cell mediated colitis. We showed that contrary to Rag2^-/-^ mice, in which adoptive transfer of naïve CD4^+^CD45RB^Hi^ T cells requires typically 6–8 weeks for the development of moderate disease, T cell-transferred DKO mice developed rapidly progressive colitis in less than two weeks. This exacerbated phenotype included rapid body weight loss, increased mucosal T cell and neutrophil influx, increased mucosal cytokine expression, increased permeability, and expansion of CD25^-^FoxP3^+^ Tregs. The latter has been reported in several autoimmune diseases such as systemic lupus erythematosus [43–45], multiple sclerosis [46], idiopathic thrombocytopenic purpura [47], as well as in a mouse model of Crohn’s-like ileitis [48]. Although the function of these cells remains controversial, their immunosuppressive potential is likely diminished due to reduced ability to scavenge IL-2 [49].

The severely exacerbated phenotype observed in DKO mice was reduced and normalized with the preventive administration of clinically relevant combination of broad-spectrum antibiotics, thus highlighting the key role of intestinal microbiota in the pathogenesis of colitis in this model. Consistent with our previous observations [27], at the onset of the study, DKO mice had a significant colonic dysbiosis with reduced microbial diversity, and significant changes in taxonomic composition. This included a reduced relative abundance of *Lactobacillaceae*, *Ruminococcaceae*, and some Clostridia, including cluster XIVa (Clostridium coccoides group) known for its immunomodulatory effects [35], decreased abundance in ulcerative colitis (UC), and associated with remission after FMT in UC patients [50]. Loss of NHE3 in DKO mice was also associated with a significant expansion of *Erysipelotrichaceae* (30.06% in DKO vs. 5.29% in Rag2^-/-^; Table 1), a *Firmicutes* clade most known for its bloom associated with high-fat diet [51, 52]. The changes in abundance and the role of *Erysipelotrichaceae* family in IBD appear to follow a different pattern. Contraction has been shown in treatment-naïve Crohn’s patients, and expansion was associated with clinical response to exclusive enteral nutrition [53, 54]. On the other hand, increased relative abundance of this family was shown in patients with colorectal cancer [55]. The expansion of *Erysipelotrichaceae* in DKO mice was primarily driven by increased relative abundance of the *Allobaculum* genus. Similar findings have been reported in experimental colitis in IL-22 deficiency model with decreased production of antimicrobial peptides RegIIIβ and RegIIIγ [41]. However, *Allobaculum* bloom was more likely related to inflammation in general than to IL-22 deficiency since compared to T-cell transferred Rag^-/-^ mice, our DKO mice had significantly higher mucosal expression of IL-22 and RegIIIβ (Fig 5).

Statistical (ANOSIM) and visual (PCoA) comparisons of unweighted UniFrac distances between gut microbiome samples indicated that NHE3 deficiency was the single most significant factor associated with microbial dysbiosis, and that despite very strong inflammatory response to the adoptive T cell transfer, within the 13 day time frame of the study, fecal microbial ecology was not significantly affected by the developing colitis. Future studies will need to address whether this strong genotype effect on the gut microbiota would translate into transmissible colitis via horizontal transfer of altered fecal microbiota from NHE3-deficient mice into NHE3-sufficient mice with genetic susceptibility to colitis. A possibility like that is supported by data from TRUC mouse model [Rag2^-/-^ x Tbx22^-/-^; [56]] or NLRP6^-/-^ mice [57]. While the question of whether the changes in microbial diversity and composition are the cause or the result of the inflammation remains open, we hypothesize that both may be true. In this scenario, inflammatory mediators (even in the absence of frank histological evidence of colitis) may lead to impaired epithelial Na^+^/H^+^ activity, which leads to changes in the gut microbiome and ultimately to exacerbated immune response to dysbiotic commensal microbiome. Our observations also indicate that in the future, approaches aimed at normalizing epithelial Na^+^/H^+^ exchange may become valuable in IBD treatment not only as means of restoring ion and fluid homeostasis, but also as means of restoring normal gut microbial ecology and controlling the immune reactivity.
